# A deep sequencing approach to estimate *Plasmodium falciparum* complexity of infection (COI) and explore apical membrane antigen 1 diversity

**DOI:** 10.1186/s12936-017-2137-9

**Published:** 2017-12-16

**Authors:** Robin H. Miller, Nicholas J. Hathaway, Oksana Kharabora, Kashamuka Mwandagalirwa, Antoinette Tshefu, Steven R. Meshnick, Steve M. Taylor, Jonathan J. Juliano, V. Ann Stewart, Jeffrey A. Bailey

**Affiliations:** 1 Uniformed Services University, 4301 Jones Bridge Road, Bethesda, MD USA; 2 Program in Bioinformatics and Integrative Biology, University of Massachusetts School of Medicine, 55 Lake Avenue North, Worcester, MA USA; 3 University of North Carolina School of Medicine, 101 Manning Drive, Chapel Hill, NC USA; 4 Ecole de Santé Publique, Université de Kinshasa, Commune de Lemba, P.O Box 11850, Kinshasa, Democratic Republic of Congo; 5 Division of Infectious Diseases and Duke Global Health Institute, Duke University Medical Center, 303 Research Drive, Durham, NC USA

**Keywords:** *Plasmodium falciparum*, Complexity of infection, Amplicon-based deep sequencing, Apical membrane antigen 1

## Abstract

**Background:**

Humans living in regions with high falciparum malaria transmission intensity harbour multi-strain infections comprised of several genetically distinct malaria haplotypes. The number of distinct malaria parasite haplotypes identified from an infected human host at a given time is referred to as the complexity of infection (COI). In this study, an amplicon-based deep sequencing method targeting the *Plasmodium falciparum apical membrane antigen 1* (*pfama1*) was utilized to (1) investigate the relationship between *P. falciparum* prevalence and COI, (2) to explore the population genetic structure of *P. falciparum* parasites from malaria asymptomatic individuals participating in the 2007 Demographic and Health Survey (DHS) in the Democratic Republic of Congo (DRC), and (3) to explore selection pressures on geospatially divergent parasite populations by comparing AMA1 amino acid frequencies in the DRC and Mali.

**Results:**

A total of 900 *P. falciparum* infections across 11 DRC provinces were examined. Deep sequencing of both individuals, for COI analysis, and pools of individuals, to examine population structure, identified 77 unique *pfama1* haplotypes. The majority of individual infections (64.5%) contained polyclonal (COI > 1) malaria infections based on the presence of genetically distinct *pfama1* haplotypes. A minimal correlation between COI and malaria prevalence as determined by sensitive real-time PCR was identified. Population genetic analyses revealed extensive haplotype diversity, the vast majority of which was shared across the sites. AMA1 amino acid frequencies were similar between parasite populations in the DRC and Mali.

**Conclusions:**

Amplicon-based deep sequencing is a useful tool for the detection of multi-strain infections that can aid in the understanding of antigen heterogeneity of potential malaria vaccine candidates, population genetics of malaria parasites, and factors that influence complex, polyclonal malaria infections. While AMA1 and other diverse markers under balancing selection may perform well for understanding COI, they may offer little geographic or temporal discrimination between parasite populations.

**Electronic supplementary material:**

The online version of this article (10.1186/s12936-017-2137-9) contains supplementary material, which is available to authorized users.

## Background

Malaria caused an estimated 429,000 deaths worldwide in 2015, with the overwhelming majority of deaths occurring in sub-Saharan Africa [1]. In regions of holoendemic malaria transmission, individuals are routinely exposed to malaria parasites and subsequently develop naturally acquired partial immunity to malaria clinical disease despite harbouring malaria parasites [2–10]. Individuals with asymptomatic or chronic malaria have been identified as important reservoirs for malaria transmission and represent a major challenge for malaria control and elimination strategies [11–15].

Early molecular studies revealed that genetically diverse *Plasmodium falciparum* strains circulate in malaria endemic regions and that this genetic heterogeneity contributes to the ability of *P. falciparum* to evade the host immune response and develop resistance to anti-malarial drugs [16–22]. It has been suggested that multiclonal malaria infections can influence clinical outcomes in a manner that is dependent on transmission intensity [23], and may negatively impact an individual’s response to anti-malarial drug treatment [24]. Further, multiclonal *P. falciparum* infections increase the likelihood of inter-strain genetic recombination during the sexual stage in the anopheline vector, resulting in the generation of genetically diverse *P. falciparum* strains and facilitating parasite evolution [25–29]. Multiclonal *P. falciparum* infections can occur either via multiple mosquito bites each with a different strain of *P. falciparum* or via a single mosquito bite containing multiple *P. falciparum* strains [4, 30, 31]. The number of distinct *P. falciparum* strains present within a single individual is defined as the complexity of infection (COI) [32]. The relationship between COI and malaria transmission intensity is complex. On one hand, recent studies have shown a positive correlation between the intensity of malaria transmission and *P. falciparum* COI, with malaria holoendemic regions typically experiencing higher *P. falciparum* COIs compared to areas with seasonal or low malaria endemicity [6, 33–39]. Thus, COI has been proposed as a method for measuring changes in malaria transmission intensity after the implementation of malaria control programmes [33, 35, 40–42]. Conversely, other studies have demonstrated a lack of correlation between malaria transmission intensity and *P. falciparum* COI [43–45]. Additional studies into the relationship between malaria transmission intensity and *P. falciparum* COI are, therefore, needed to better understand the relationship between malaria parasite genetic diversity and transmission dynamics and the potential utility of COI as a measure of change in malaria prevalence.

Several genetic tools and strategies have been employed to detect multiclonal *P. falciparum* infections, including targeting size polymorphisms of the merozoite surface proteins (MSP1, MSP2) and GLURP [5, 8, 46–49]. Some PCR based methods rely on DNA sequence length polymorphisms, which can be visualized via gel or capillary electrophoresis and the COI defined as the number of distinct bands present. However, these methods lack the sensitivity to identify distinct *P. falciparum* strains that differ by only a few nucleotides in length or that contain single nucleotide polymorphisms (SNPs). Also, these methods have poor sensitivity in terms of detecting less abundant strains [50–53], and differing methods can result in high variability in the number of strains detected between laboratories [54]. Novel approaches based on DNA deep sequencing technologies provide increased capabilities to detect minor variant *P. falciparum* strains as well as discriminate SNPs and small indels. These deep sequencing technologies provide a more accurate determination of the COI within an individual or population thereby improving subsequent population genetic analyses [4, 6, 34, 50, 55, 56].

In the Democratic Republic of Congo, malaria is a leading cause of morbidity and mortality with over 95% of malaria infections due to *P. falciparum* [57]. The DRC Ministry of Health estimates that 97% of the population in the DRC live in areas where malaria transmission occurs 8–12 months out of the year [57]. The 2007 DRC demographic and Health Survey (DHS) and subsequent studies reported over one-third (33.5%) of adults (15–59 years) were positive for malaria by real-time PCR (qPCR) [58, 59]. Several studies have explored the complex malaria spatial epidemiology and population genetics in the DRC [59–67]. For instance, a recent spatial and genetic analysis revealed *P. falciparum* parasite populations are dispersed across seven geographical areas, likely due to movement of human populations between provinces in the DRC and the region [61]. Additionally, Taylor et al. report spatial and genetic clustering of *P. falciparum* sulfadoxine drug resistance between western and eastern DRC [65]. Further studies to examine *P. falciparum* haplotype diversity are, therefore, warranted to inform malaria control strategies and to monitor changes in malaria parasite population structure in response to malaria control efforts in the DRC.

In this study, a PCR amplicon-based deep sequencing approach was utilized to target the extensive allelic diversity of the *P. falciparum* apical membrane antigen 1 (*pfama1*) gene in order to (1) examine the relationship between *P. falciparum* COI and *P. falciparum* prevalence as determined previously by real-time PCR [59], (2) to investigate the *P. falciparum* population genetic structure at both the individual and population level in the DRC, and (3) to explore AMA1 amino acid frequencies and potential selection pressures between geographically distinct malaria parasite populations in the DRC and Mali. The authors hypothesized that *P. falciparum* COI would be positively correlated with *P. falciparum* prevalence in a region, and that similar *pfama1* haplotypes would be identified at the individual and population level in the DRC and Mali. In order to investigate *pfama1* diversity at both the individual and population level, individual samples (representing a malaria infection in a single person), and pooled samples (representing population cluster samples) were targeted in this study. Pooling samples is a cost-effective approach to amplicon-based deep sequencing as it reduces the number of PCR reactions and library preparations, and this pooled approach has been utilized in several malaria population genetic studies [68–71]. This dual sample type (individual and population cluster) approach allows for the examination of COI using the individual samples and also powers spatial population genetic analyses combining the individual samples and the pooled population cluster samples.

Overall, a total of 77 unique *pfama1* haplotypes were identified across DRC provinces. The vast majority of individual malaria infections were polyclonal (COI > 1), and no correlation was found between COI and malaria prevalence at sites/regions. Population genetic analyses revealed extensive genetic diversity of *P. falciparum* parasites based on the *pfama1* gene and similar amino acid frequencies between malaria parasite populations in the DRC and Mali. Herein, this manuscript highlights the utility of combining individual and pooled amplicon-based deep sequencing methods for population genetic analyses layered onto the infrastructure and sample collection process of a routine Demographic and Health Survey. This manuscript also describes the spatial and genetic diversity of *pfama1* haplotypes circulating in the DRC and Mali to improve the understanding of malaria transmission dynamics that could potentially inform future malaria control and elimination efforts in the region.

## Methods

### Ethics statement

Participants included in the Demographic and Health Survey (DHS) provided verbal informed consent as described previously [59]. Study enrollment and blood sample collection protocols were approved by the Ethics Committees of the institutions involved in the DHS and sample collection including Macro International, the School of Public Health of the University of Kinshasa, and the Institutional Review Board of the University of North Carolina.

### Democratic Republic of Congo Demographic Health Survey Sample Collection

The 2007 Democratic Republic of the Congo Demographic and Health Survey (DHS) was conducted to collect health indicator data from across the DRC. Within 300 clusters, survey teams went from house to house and enrolled women aged 15–49 years, and men aged 15–59. The age distribution was constant across sites. The survey in urban Kinshasa occurred during the rainy season (January 31–March 8, 2007). The remainder of the country was surveyed during the dry season (May–August, 2007) [58, 60]. Genomic DNA was extracted from dried blood spot (DBS) samples on filter paper for malaria species-specific 18S ribosomal RNA based qPCR detection of *P. falciparum, Plasmodium ovale,* and *Plasmodium malariae* parasites [59]. The samples used herein were randomly selected from samples obtained during the 2007 DHS.

**Fig. 1:**
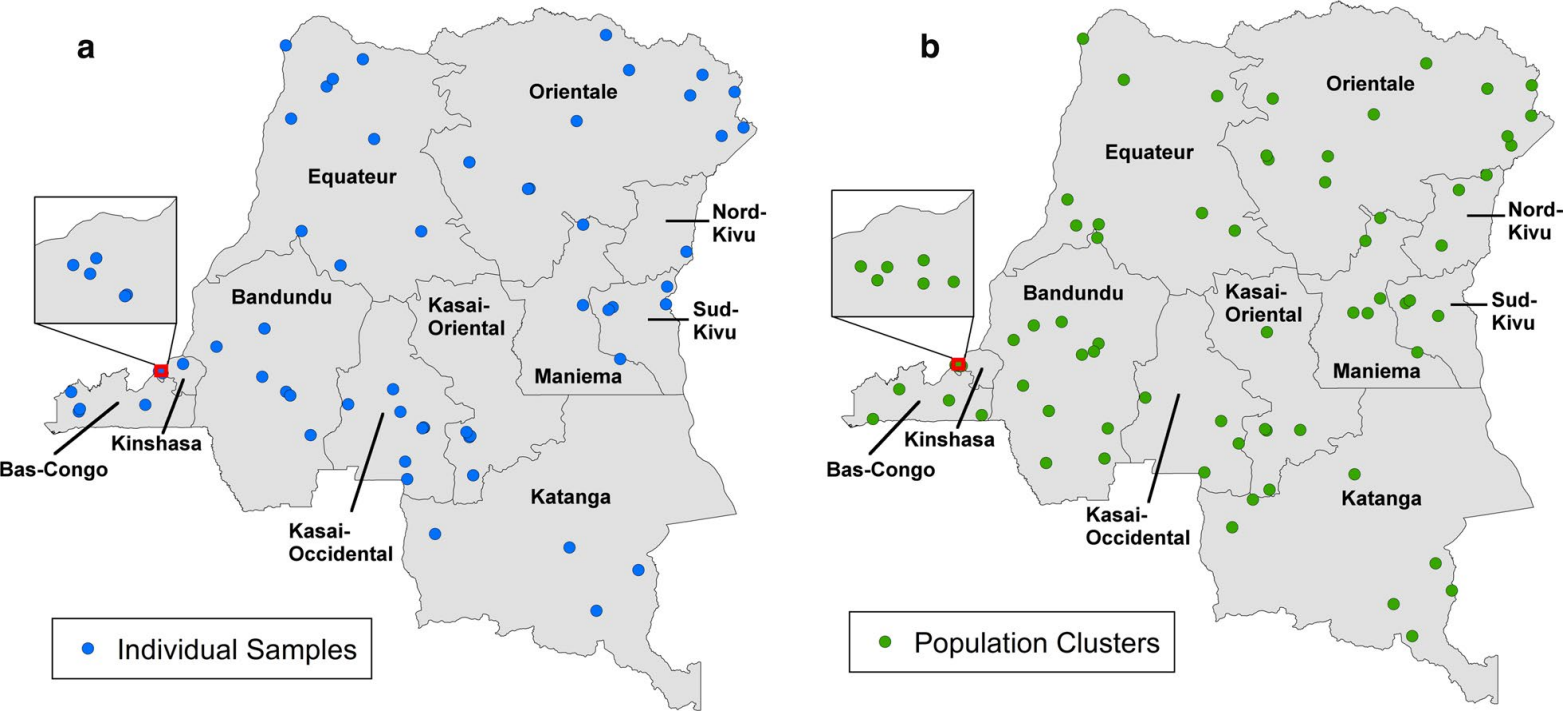
Individual (**a**) and population cluster (**b**) samples with analysable sequence reads locations. Blood samples were collected from all seven provinces including Kinshasa (box inset). Individual samples (blue circles, n = 79) represent genomic DNA isolated from a single person and population cluster samples (green circles, n = 73) are pooled genomic DNA samples from multiple (2–25) people

In the present study, 115 individual samples positive for asymptomatic *P. falciparum* infection were identified based on the following criteria: (1) positive for *P. falciparum* and negative for *P. ovale* and *P. malariae* by species-specific qPCR and (2) geographically representative of the eleven DRC provinces. Eighty-four population cluster samples that represented pooled asymptomatic *P. falciparum* samples of 2–25 individuals were also chosen based on geographical proximity to the individual samples (Additional file 1). Individual and population cluster samples were selected in order to compare parasite haplotypes from individual people (Fig. 1a) and the parasite population at large (Fig. 1b) from all DRC provinces. The presence of *P. falciparum* parasites was further confirmed for all individual samples based on the detection of *P. falciparum* *lactate dehydrogenase* (*pfldh*) gene by qPCR as described [72].

### Amplicon-based deep sequencing of the *P. falciparum* apical membrane antigen 1 (*pfama1*) gene

**Fig. 2:**
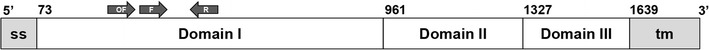
Schematic of the *Plasmodium falciparum* *apical membrane antigen 1* (*pfama1*) gene. The first 72 nucleotides contain the signal sequence (ss) followed by Domain I (nucleotide 73–960), Domain II (nucleotide 961–1326), Domain III (nucleotide 1327–1638), and the transmembrane domain (tm, nucleotide 1639–1869). The heminested primer binding sites (OF = Ama1OF, F = Ama1F, R = Ama1R) are indicated by arrows located in Domain I. Nucleotide sites are based on the 3D7 reference strain (GenBank XM_001347979.1)

To increase assay sensitivity, heminested primers were designed using Primer3 [73] to amplify a region of the *pfama1* gene (GenBank Reference XM_001347979.1) (Fig. 2). The first round PCR primers, Ama1OF and Ama1R, and the second round PCR primers, Ama1F and Ama1R primers, amplify a 266 base pair (bp) and 236 bp region, respectively (Additional file 2). In order to pool PCR amplicons during sequencing library preparation, a 10-nucleotide Multiplex Identifier (MID) barcode sequence was added to the 5′ end of the Ama1F primer [74] (Additional file 2). PCR amplicons were visualized on 1% agarose gels stained with ethidium bromide and purified via the PureLink Pro 96 PCR Purification Kit (Invitrogen, Carlsbad, CA). PCR amplicon concentration was determined in duplicate and averaged using the Quant-iT PicoGreen dsDNA Assay Kit (Invitrogen, Carlsbad, CA) according to manufacturer’s instructions.

Purified PCR amplicons containing barcodes were pooled into sets (up 24 amplicons each based on the 24 unique MIDs) at equal concentration. Each pool was then used to generate a sequencing library with a unique index to allow for the unambiguous identification of the sequences for every amplicon. Specifically, ten nanograms of pooled purified PCR amplicons was ligated with the appropriate index and the DNA concentration of each indexed amplicon pool was determined using the Agilent High Sensitivity D1000 ScreenTape Assay on the 2200 TapeStation (Agilent Technologies, Santa Clara, CA) according to manufacturer’s instruction in order to confirm equal DNA concentrations across the 18 indexed amplicon pools. Six sequencing control samples were run as template in duplicate with the *pfama1* PCR assay and included in downstream sequencing library preparations. Sequencing control samples contained *P. falciparum* DNA from V1S, RO33, Dd2, 7g8, and K1 strains at 5, 10, 15, 30, and 40 percent, respectively (BEI Resources/MR4, Manassas, VA).

Ion Torrent library preparation was conducted following the “Preparing Short Amplicon (< 350) Libraries Using the Ion Plus Fragment Library Kit” manual (Life Technologies, MAN0006846, revision 3.0) for each of the 18 indexed PCR pools. DNA concentrations of the 18 resulting libraries were determined using the Agilent High Sensitivity D1000 ScreenTape Assay according to manufacturer’s protocol. Equal concentrations of each library were pooled and split across two Ion 318 Chips (Life Technologies, Carlsbad, CA) utilizing 400 bp chemistry on the Ion Torrent PGM platform (Life Technologies) at the University of North Carolina Chapel Hill Microbiome Core Facility. Deep sequence data extraction, processing, and analyses were performed using the SeekDeep targeted amplicon bioinformatics pipeline [75–79]. A workflow diagram is provided to outline the methods and provide additional details (Additional file 3).

In order to compare AMA1 amino acid frequencies between the DRC and Mali, 506 *pfama1* sequences (GenBank FJ898536–FJ899041) previously published by Takala et al. were retrieved [80]. *Pfama1* sequences from Mali were trimmed and aligned to *pfama1* sequences from the DRC using Geneious (v 9.1.5) [81].

### Population genetics analyses

Molecular population genetic analyses were conducted using MEGA version 7 [82] and DnaSP (v5.10.1) [83, 84]. Standard nucleotide and haplotype diversity calculations weighting pools by the number of contained individuals [85] were performed in Python. Population pairwise (F_ST_) comparisons were calculated between DRC provinces using the Analysis of Molecular Variance (AMOVA) tool in the Arlequin (v3.5.2.2) population genetics data analysis program [86]. Network (v5.0.0.1), DNA Alignment (v1.3.3.2), and Network Publisher (v2.1.1.2) add-ons were used to generate a median-joining (MJ) network diagram in order to visualize phylogenetic relationships between *pfama1* haplotypes [87]. Isolation by Distance (IBD) analysis was performed using the Mantel Test in GenAlEx (v.6.503) [88, 89].

### Statistical analyses and data visualization

Statistical analyses were performed in GraphPad Prism (v6), SPSS (v22), R [90], and Microsoft Excel. ArcGIS (ESRI, v.10.4.1.5686) was used to generate maps and the DRC province boundary map was obtained from the DHS Programme Spatial Data Repository [91]. All permutation testing used 10,000 replicates.

## Results

### Amplicon-based deep sequencing of individual and population cluster samples

Real-time PCR (qPCR) was performed for all 115 individual samples to confirm the presence of *P. falciparum* based on the *lactate dehydrogenase* (*pfldh*) gene. Conventional PCR based on the *pfama1* gene was performed on all individual samples regardless of *pfldh* qPCR results and on all geographical cluster samples (n = 84). Table 1 summarizes the results of the *pfldh* qPCR and *pfama1* PCR for both sample types.

**Table 1:** Summary of DRC individual samples and population cluster samples PCR and amplicon deep sequencing results

Sample type	Number of samples	No. of *pfldh* qPCR positive samples (%)^a^	No. of *pfama1* PCR positive samples (%)	No. of samples with analysable deep sequence reads (%)^b^
Individual	115	99 (86.1)	81 (70.4)	79 (68.7)
Population cluster	84	–	79 (94.0)	73 (86.9)

^a^Concentrations of *pfldh* based on qPCR standard curve analysis ranged from less than 0.1 ng/ml to over 1000 ng/ml for individual samples

^b^The number of samples with analysable deep sequence reads was determined using the SeekDeep targeted amplicon analysis pipeline criteria and a 2.5% minimum haplotype frequency cutoff requirement for inclusion in analysis. The SeekDeep analysis pipeline excludes reads based on missing barcodes, short reads (< 50 bp), and poor quality/chimeric reads

A total of 11,511,315 *pfama1* deep sequencing reads were obtained using the Ion Torrent PGM platform. Using the SeekDeep targeted amplicon analysis pipeline, reads with missing barcodes, short reads (< 50 bp), poor quality, and chimeric reads were filtered out, resulting in 4,879,911 remaining reads. The IonTorrent PGM platform is based on the “sequencing by synthesis” principle (detecting H ion release on a semiconductor matrix when a base is added during synthesis), which can result in variable quality reads particularly within homopolymer repeats. Thus, sequencing read quality varies in the proportion of reads that are poor quality or truncated and is mainly dependent on the quality of the input library as well as the specific run rather than one sequencing platform over another. These sequencing reads were subsequently de-multiplexed (separated by input amplicon based on index and MID), clustered according to samples and replicates, and haplotypes estimated using a 2.5% minimum haplotype frequency cutoff. Based on these criteria, a total of 3,754,497 reads was obtained for downstream haplotype analysis. Analysable deep sequencing reads of the target *pfama1* region were generated for 79 (68.7%) of the individual samples, 73 (86.9%) of the population cluster samples, and six sequencing control samples. Analysis of the six sequencing control samples revealed similar haplotype frequencies between the expected haplotype percentage and the actual haplotype percentage determined by the SeekDeep targeted amplicon analysis pipeline (Additional file 4), demonstrating the sensitivity of a targeted amplicon based deep sequencing approach to detect mixed haplotype infections. No false haplotypes (i.e. haplotypes that were not included in the control template) were detected from the control sample sequencing reads.

Overall, a total of 77 unique *pfama1* haplotypes from both the individual samples and population cluster samples were identified (Table 2; Additional files 5, 6). A total of 60 *pfama1* haplotypes were identified in individual samples and 55 haplotypes were identified in the population cluster samples (Table 2; Additional file 6). Thirty-eight *pfama1* haplotypes were shared between the individual and population cluster samples and 25 most frequent haplotypes were detected in both confirming their general equivalence (R = 0.71) (Additional file 6). Twenty-two and seventeen *pfama1* haplotypes were unique to the individual samples and population cluster samples, respectively. Only relatively low frequency haplotypes in the population were not detected in both individual and pooled samples (frequency average 0.1%; maximum 1.0%) (Additional file 6).

**Table 2:** Summary DRC population genetic data based on sample type

	Number of samples	Number of haplotypes	Mean COI	# Polymorphic sites (S)	Nucleotide diversity (π)	Haplotype diversity (Hd)
Total	152	77	–	33	0.04458	0.9552
Individual samples	79	60	2.38	32	0.04378	0.9550
Population cluster samples (#Ind./pop)^a^	73 (821)	55	–	20	0.04456	0.9536

COI: Complexity of infection

^a^Number of individual samples within the population cluster samples

**Fig. 3:**
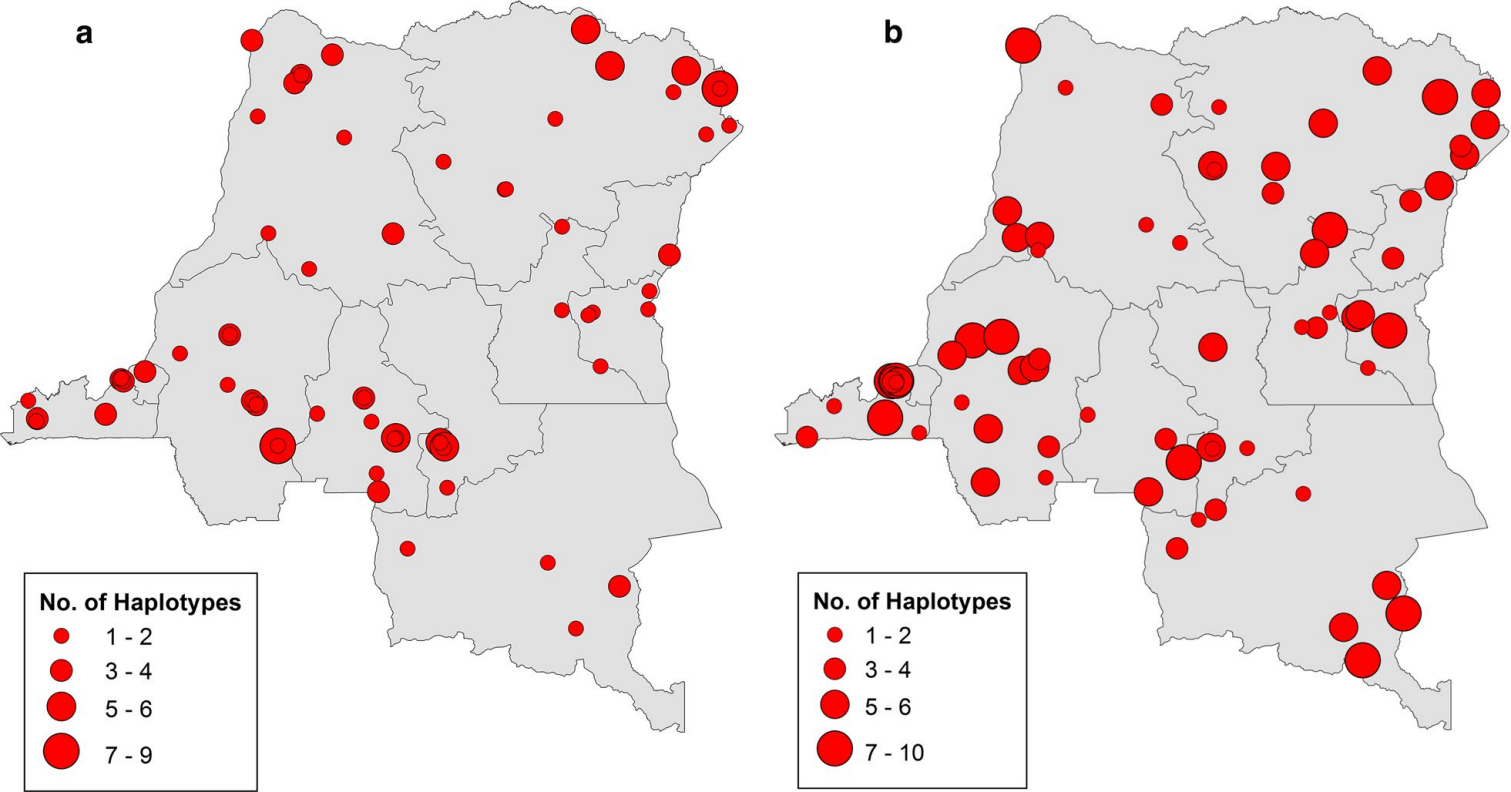
Haplotype frequencies in individuals (**a**) and population clusters (**b**) based on geographical location. Circle size represents the number of unique *pfama1* haplotypes in a particular location. Georeferencing data was unavailable for four individual samples and three population cluster samples

**Fig. 4:**
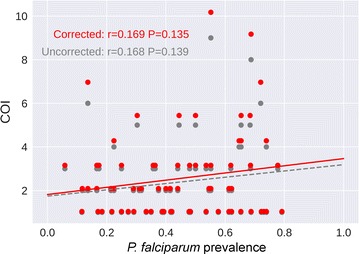
Comparison of *Plasmodium falciparum* complexity of Infection (COI) and prevalence. The relationship between the observed (uncorrected, in grey) *P. falciparum* COI from individual samples and *P. falciparum* prevalence by qPCR showed a small positive correlation that was not significant (Pearson coefficient of correlation, r = 0.168; p = 0.139). A permutation model was used to account for strains that share the same *pfama1* haplotype and average corrected COI values (red) are shown. No significant correlation between corrected COI and *P. falciparum* prevalence by qPCR was observed (Pearson coefficient of correlation, r = 0.169, p = 0.135)

The majority of *P. falciparum* infections in individual samples were polyclonal (64.5%) defined as a COI > 1 (Fig. 3a). The mean COI for individual samples was 2.38 and ranged from 1 to 9 haplotypes (Table 2). Not surprisingly, given they represent multiple patient samples, 84.9% of the population cluster samples were polyclonal (Fig. 3b). Comparison of the demographic characteristics between the sample types with analysable sequence reads revealed the pooled subjects tended to be slightly younger, to live in rural areas, and to be male than the subjects tested individually (Additional file 7). In order to explore the relationship between COI and malaria transmission intensity in the DRC, COI from individual samples were compared to *P. falciparum* prevalence obtained via real-time PCR from the 2007 DHS samples reported in a separate study [59]. As shown in Fig. 4, there was a non-significant weak linear trend of increasing COI with prevalence but the overall variance was high with little accounted for by this model (Pearson coefficient of correlation, r = 0.168, p = 0.139 by permutation) (Fig. 4, grey). A nonparametric Spearman rank correlation was also performed and demonstrated no significant relationship between observed COI and *P. falciparum* prevalence by qPCR (r_s_ = 0.126, p = 0.268 by permutation). Difference in observed COI and bifurcated prevalence based on the mean (0.4413) was also tested. These high and low prevalence groups had a mean of 2.75 and 2.00, respectively, with suggestive significance (Wilcoxon two sample test, p = 0.0861 and p = 0.0353 by permutation).

*Pfama1* amplicon deep sequencing, like all single locus methods, will underestimate the true COI when strains share the same haplotype. A permutation model was utilized to correct for COI when strains share the same *pfama1* haplotype. Based on the observed *pfama1* haplotype frequencies, the probability of true COI of 2, 3, 4, 5, 6, 7, 8, 9, and 10 being undercalled (missing one or more strains) is 4.5, 12.7, 24, 37, 50, 62, 73, 82 and 88 percent, respectively. While these values demonstrate that the highest COIs are usually underestimated, these high COIs represent a low proportion of the observed samples in this study. Using both the probability of undercalling and the observed distribution, the corrected COIs were simulated and the average correction plotted (Fig. 4, red). Compared to the original uncorrected COI, there was minimal change in the parametric correlation (Pearson coefficient of correlation, r = 0.169, p = 0.135 by permutation) and no change in the nonparametric correlation (Spearman, r = 0.126, p = 0.268 by permutation). The mean corrected COI showed an increased spread of 2.98 versus 2.12 between the high and low prevalence groups, respectively. Despite this increased difference, the *p* value increased slightly (p = 0.0398 by permutation) due to increased variance resulting from modeling the imprecision of the COI measurements.

### Population genetic analyses

Population genetics analysis methods were utilized to explore *pfama1* haplotypes from all sample types as well as between individual samples and population cluster samples (Table 2). Overall, 33 polymorphic sites (S) from 77 *pfama1* haplotypes and high haplotype diversity (Hd) were found in both sample types (Table 2). Comparison between individual and population cluster samples show similar haplotype diversity (Hd) and nucleotide diversity (π). This was supported by the lack of a statistical difference between the two types of sampling at the province level (Wilcoxon Rank Sign Test, p = 0.16 for Hd; p = 0.60 for π). However, there were fewer polymorphic sites (S) in the population cluster samples compared to the individual samples (Table 2) and these additional sites represent low prevalence rare variants within the population. This emphasizes that pooled samples should not be utilized for analyses that target or depend on the assessment of low frequency variants.

The population genetic data were examined based on DRC province for both individual and population cluster samples (Table 3). Both sample types showed similar numbers of polymorphic sites, nucleotide diversity, and haplotype diversity across the 11 provinces. However, Bas Congo appeared to differ from other provinces with the lowest haplotype diversity (0.568) and nucleotide diversity (0.0277). The overall frequencies were tested for significant outliers. Bas Congo was identified as an outlier in terms of haplotype diversity (p = 0.004; Dixon’s Q-test) but not in terms of nucleotide diversity (p = 0.6). However, this overall deviation was only supported by the pooled samples (n = 30; p = 0.004) and not the individuals (n = 4; p = 0.6). Given this difference and that this province was less deeply sampled than on average it is not clear if this is a significant deviation.

**Table 3:** Summary population genetic data based on sample type and province

	Number of samples			Number of haplotypes			# Polymorphic sites (S)			Nucleotide Diversity (π)			Haplotype diversity (Hd)		
	All	Ind.^a^	Pop^b^ (#Ind./pop)^c^	All	Ind.	Pop.	All	Ind.	Pop.	All	Ind.	Pop.	All	Ind.	Pop.
Bandundu	23	11	12 (177)	35	20	29	20	19	18	0.0404	0.0401	0.0402	0.925	0.884	0.922
Bas-Congo	8	4	4 (30)	15	7	10	18	17	17	0.0278	0.0306	0.0197	0.569	0.769	0.451
Equateur	19	11	8 (112)	35	21	23	22	21	19	0.0434	0.0434	0.0429	0.921	0.913	0.908
Kasai-Occidental	13	9	4 (38)	25	14	16	20	18	17	0.0405	0.0396	0.0400	0.909	0.876	0.886
Kasai-Oriental	16	8	8 (107)	30	19	20	20	20	18	0.0457	0.0437	0.0455	0.8740	0.842	0.866
Katanga	10	4	6 (69)	22	6	19	20	13	19	0.0396	0.0344	0.0392	0.906	0.724	0.899
Kinshasa	13	7	6 (39)	28	13	23	21	18	18	0.0419	0.0388	0.0411	0.888	0.836	0.86
Maniema	10	4	6 (54)	21	6	16	21	14	18	0.0341	0.0319	0.0335	0.879	0.758	0.863
Nord-Kivu	5	2	3 (15)	12	5	10	18	14	17	0.0386	0.0389	0.0364	0.750	0.714	0.701
Orientale	26	14	12 (147)	40	27	27	21	21	19	0.0440	0.0403	0.0442	0.9202	0.909	0.91
Sud-Kivu	9	5	4 (33)	19	6	16	19	15	18	0.0315	0.0348	0.0291	0.834	0.731	0.791

^a^Individual samples

^b^Number of population cluster samples in the province

^c^Number of individual samples that make up the pooled population cluster samples

**Fig. 5:**
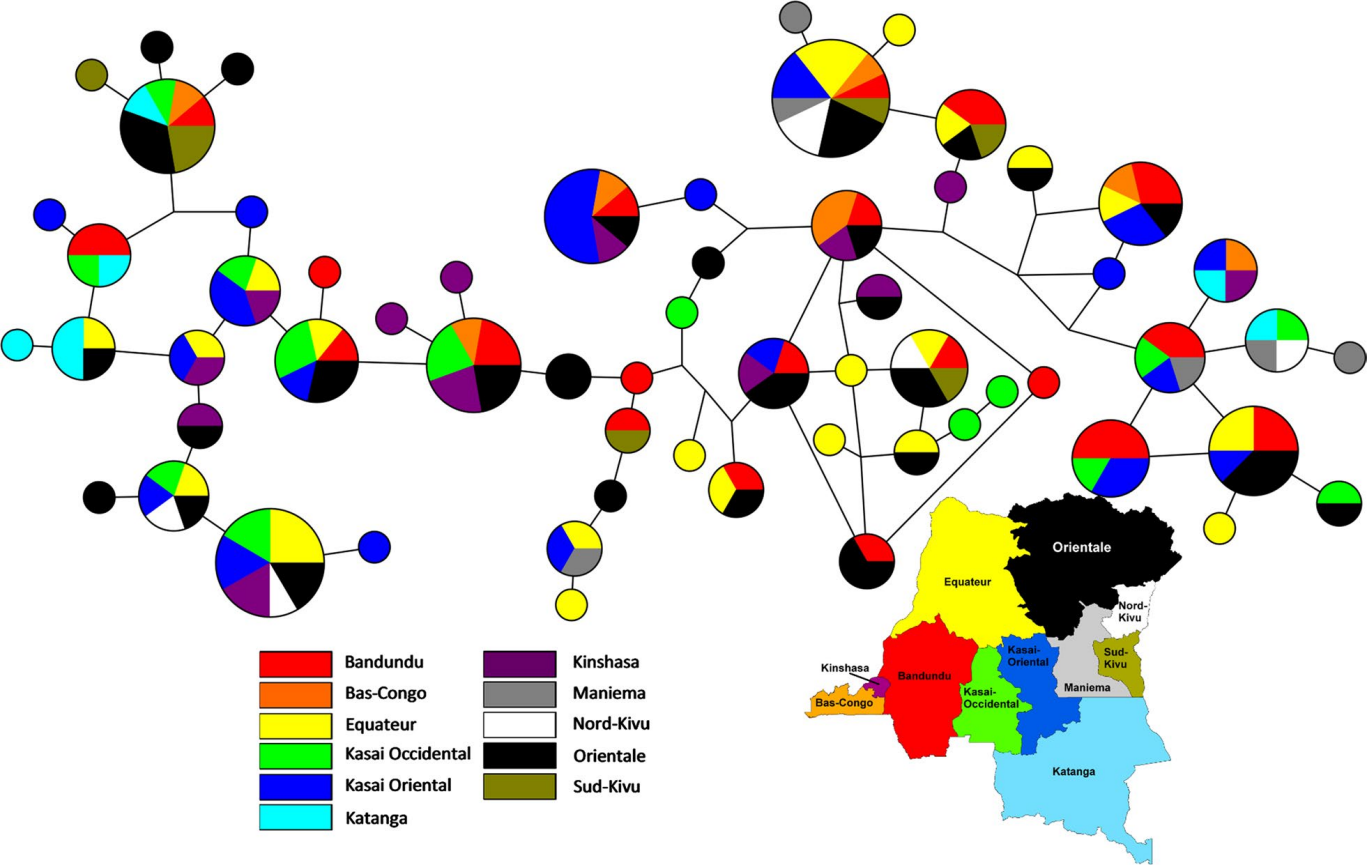
Median-joining Network Diagrams of *pfama1* haplotypes from individual samples. Each circle represents a different haplotype, the size of the circle reflects the number of individual samples with that haplotype, and the colors indicate province

To further explore haplotype diversity between DRC provinces, population fixation index (F_ST_) was determined between DRC provinces from haplotypes identified in both sample types (Table 4). Overall, the average F_ST_ value is 0.0008, ranging from 0 to 0.011656. Low F_ST_ values between provinces indicate *pfama1* haplotypes are panmictic and, therefore, not isolated based on province in the DRC. Isolation by distance (IBD) analysis of individual samples showed no correlation of genetic distance and spatial distance (R^2^ = 7.7 × 10^−5^), indicating that genetically similar *pfama1* haplotypes are not found closer together spatially. A Median-Joining Network Diagram was also constructed using the 60 haplotypes found in individual samples to examine whether related haplotypes are spatially clustered in the DRC (Fig. 5). There was no clustering of related *pfama1* sequences based on DRC province, suggesting that genetically related *pfama1* haplotypes do not appear to be spatially restricted in the DRC.

**Table 4:** Population pairwise Fst comparisons between provinces

	Bandundu	Bas Congo	Equateur	Kasai Occidental	Kasai Oriental	Katanga	Kinshasa	Maniema	Nord Kivu	Orientale	Sud Kivu
Bandundu	0										
Bas-Congo	0	0									
Equateur	0.00138	0	0								
Kasai-Occidental	0.00085	0	0	0							
Kasai-Oriental	0.00255	0	0	0	0						
Katanga	0.00178	0	0.00066	0	0	0					
Kinshasa	0	0	0	0	0	0	0				
Maniema	0	0	0	0	0	0.00713	0.00196	0			
Nord-Kivu	0.0117	0	0	0	0	0.0111	0.00149	0.00085	0		
Orientale	0	0	0	0.00123	0.0017	0.00202	0	0	0	0	
Sud-Kivu	0	0	0	0	0.00205	0	0	0	0	0	0

### Comparison of *pfama1* amino acid frequencies between geographically divergent malaria populations in the DRC and Mali

**Fig. 6:**
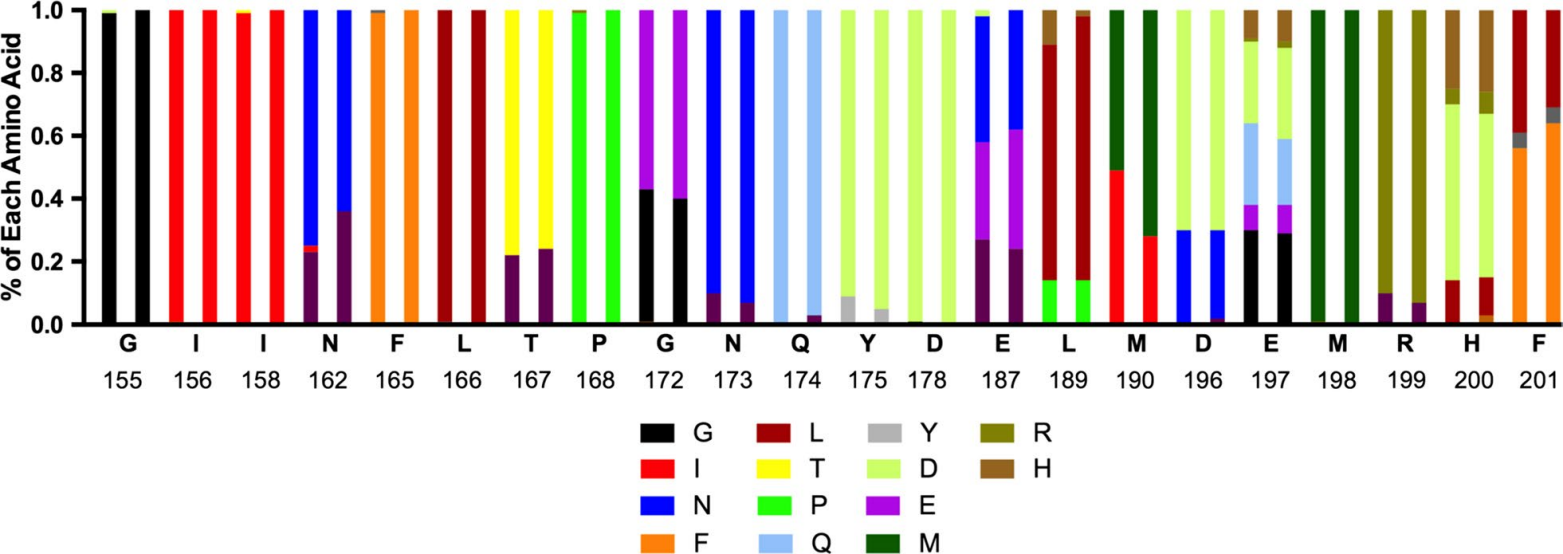
Comparison of AMA1 amino acid frequencies at variable sites between malaria populations in the DRC and Mali. The reference 3D7 amino acid sequence and corresponding amino acid position number are shown on the x-axis (GenBank XP_001348015.1). For each amino acid residue, the first bar represents the amino acid frequencies from the DRC *pfama1* haplotypes and the second bar represents the amino acid frequencies from *pfama1* haplotypes in Mali [92]. The amino acid frequency within this region of AMA1 is similar between the two geographical locations

In order to explore the heterogeneity in amino acid frequencies between malaria populations from disparate geographical locations, the 77 *pfama1* amino acid sequences from the DRC were compared to 506 previously published *pfama1* sequences from Mali (FJ898536–FJ899028) [92]. Trimming the Mali *pfama1* sequences to match the 162 bp region sequenced in the DRC samples resulted in 58 distinct Mali *pfama1* sequences. The trimmed sequences were then aligned with the DRC *pfama1* sequences and 32 (55%) of the DRC *pfama1* sequences were found to be 100% identical over a 162 bp region to the Mali *pfama1* sequences previously identified. Nucleic acid sequence identity between the DRC and Mali *pfama1* sequences ranged from 91.4 to 100%. Analysis of the DRC and Mali *pfama1* sequence heterogeneity at the amino acid level (Fig. 6) revealed highly similar amino acid frequencies between the two parasite populations despite both geographical and temporal separation suggesting balancing selection at a continental scale.

## Discussion

In this study, an amplicon-based deep sequencing was utilized to investigate the diversity of *pfama1* genes from asymptomatic malaria infections at both the individual and population cluster level from across the DRC and in Mali. Overall, a total of 77 unique *pfama1* haplotypes were identified and the majority of individual infections in the DRC were polyclonal (64.5%). Population genetic analyses revealed *pfama1* haplotypes are not isolated based on distance or province within the DRC. These results align with a previous study in the DRC, which found a lack of spatial restriction of malaria parasite populations. This diversity, however, may not be due to the extensive movement of *P. falciparum* parasites with their human hosts between provinces and neighbouring countries [61]. Rather, more likely, potential explanations for the extensive *pfama1* haplotype diversity identified in the DRC in this study include human host immune selection that maintains the antigenic diversity of *pfama1* (balancing selection) and spatially restrictive protein–protein interactions [92, 93].

In order to more fully explore *pfama1* diversity between geographically divergent malaria endemic regions, haplotype frequencies were compared at the amino acid level in parasite populations from the DRC and Mali. Highly similar amino acid frequencies were observed between parasite populations in the DRC and Mali (Fig. 6), suggesting analogous selective pressures could be maintaining *pfama1* haplotype diversity between the two regions across the continent more so than parasite movement. A previous study to investigate the diversity of the circumsporozoite protein (CS), another hypervariable surface antigen, also showed shared amino acid frequencies between two geographically separated malaria parasite populations [93]. Highly diverse regions under balancing selection, such as AMA1, while excellent markers for COI, may therefore be poorly suited to discriminate geographically distinct malaria parasite populations or serve as a marker for malaria parasite diversity.

In contrast to several recent studies [6, 33–36], this study found a minimal positive correlation between COI and malaria prevalence that was not significant (Fig. 4). While additional samples could have increased the power in this study, other studies have also reported no correlation between COI and *P. falciparum* prevalence [43–45]. Potential explanations for these discrepancies include differing methodologies for detection of *P. falciparum* strains and varying malaria transmission intensity by region. Previous studies that reported significant correlations between COI and malaria prevalence typically compare low and high malaria transmission areas [34–37, 39]. This study was conducted in the DRC, which experiences high malaria transmission year round. Therefore, the lack of a significant association between COI and *P. falciparum* prevalence in this study compared to other studies could be because due to the high stable malaria transmission across the DRC. Additional research studies including larger sample sizes and additional markers are needed to further explore the potential relationship between COI and malaria prevalence and how population diversity indices could be utilized to monitor changes in malaria transmission intensity in the DRC and other malaria endemic regions. However, given the wide variance observed in the correlation between COI and prevalence, it may not be a reliable surrogate in differentiating malaria transmission levels within the DRC.

Deep sequencing technologies have enhanced ability to detect low frequency, minor variant *P. falciparum* haplotypes and characterize malaria COI from a variety of sample types including dried blood spots [4, 6, 34, 50, 55, 56]. Amplicon-based deep sequencing was utilized in this study to detect polyclonal *P. falciparum* infections for several reasons, including its cost-effectiveness compared to whole genome sequencing and the ability to utilize barcoding and pool several dozen samples thereby increasing sample size. The SeekDeep bioinformatics pipeline is designed for analysis of haplotype frequency from amplicon-based deep sequencing data and has been used successfully in several studies investigating malaria population genetics globally [76, 78, 79].

*Pfama1* was chosen for amplicon-based deep sequencing based on several factors. First, *pfama1* is a highly polymorphic gene, containing several single nucleotide polymorphisms (SNPs), likely maintained via balancing selection due to immune pressure in the human host [80, 94–96]. Previous studies in malaria endemic regions have identified over 60 polymorphic sites within *pfama1* [96–99]. Similarly, sequencing of human samples from a malaria endemic region in Mali identified over 200 unique *pfama1* haplotypes [80]. The *P. falciparum* AMA1 antigen is also a highly-studied malaria vaccine antigen candidate. Vaccine studies have demonstrated that AMA1 based vaccine protection against clinical malaria is extremely strain-specific and, therefore, a clear understanding of AMA1 diversity is critical to develop an effective malaria vaccine based on this polymorphic antigen [100–105]. The results from this study provide further evidence of the extensive heterogeneity of *pfama1* haplotypes in the DRC and surrounding malaria endemic regions.

This study has several important limitations that may have restricted the ability to detect minor variants and calculate COI in the malaria parasite population circulating in the DRC. These limitations include: possible *pfama1* sequence polymorphisms in primer binding sites, malaria parasite nucleic acid degradation stored on dried blood spots, and *pfama1* haplotype frequency below the limit of detection of the PCR assay or 2.5% cut off for sequencing analysis. In addition, this study focused on a subset of asymptomatic malaria samples collected as part of the 2007 DHS in the DRC. The inclusion of more malaria positive samples, including symptomatic as well as asymptomatic malaria infections, would provide a more comprehensive description of the *P. falciparum* population genetic structure in the DRC. Another potential limitation is that this study targeted a region in the highly polymorphic *pfama1* gene as surrogate for the entire *P. falciparum* genome. As such, the true genetic heterogeneity of *P. falciparum* parasites circulating in the DRC is underestimated. Further, as the number of polymorphic sites (S) was unexpectedly higher in the individual samples compared to the pooled population cluster samples, it is important to note that pooled sampling likely missed some variants occurring at low frequency within one or a few individuals within the population (Table 2). As such, it is critical to consider whether samples were pooled prior to amplicon-deep sequencing when designing studies to detect low frequency variants and for cross comparisons between individuals and pools while choosing statistics minimally influenced by rare variants or haplotypes, particularly in low malaria prevalence areas. However, targeted deep sequencing shows great improvement in COI estimates over traditional methods [50], particularly for *pfama1* given its high 0.95 haplotype diversity. To account for the chance of strains sharing the same AMA1 haplotype, a permutation-based model incorporating undercall probability was used to simulate corrected COIs (Fig. 4). Given the high heterozygosity of *pfama1* and the observed COIs, the corrections showed minimal differences compared to the observed (uncorrected) COI results (Fig. 4). This would not be the case if the average COIs in this study were higher, as COIs > 5 were estimated to be undercalled for the majority of observed measures. As deep sequencing technologies become increasingly more cost effective and less labour-intensive, future studies targeting *P. falciparum* strain diversity in malaria endemic regions could include whole genome deep sequencing.

## Conclusion

This study describes the use of amplicon-based deep sequencing for the detection and relative quantification of *P. falciparum* haplotypes and characterization of COI in the DRC and the spatial epidemiology and population genetic structure of malaria parasites from both individual and population cluster samples across eleven DRC provinces. Highly similar AMA1 amino acid frequencies between parasite populations were identified in the DRC and Mali, suggesting analogous selective pressures maintain *pfama1* diversity in geographically divergent locations and therefore limit the use of *pfama1* as a marker to discriminate parasite populations (or other markers known to be under balancing selection). Given the *P. falciparum* recent speciation bottleneck and limited diversity compared to other species, selection of more appropriate genetic markers of diversity may be a challenge. Sensitive detection methods, such as amplicon-based deep sequencing, can improve the understanding of malaria strain diversity as it relates to potential malaria vaccine antigen candidates and monitor for changes in parasite genetic diversity.

## Additional files

**Additional file 1.** Population cluster sample characteristics. Asterisks (*) indicate population cluster samples that failed initial *pfama1* PCR amplification (n=5). Diamonds (♦) indicate a population cluster sample that was not successfully sequenced (n=6).

**Additional file 2.** *Pfama1* heminested primer sequences and Multiplex identifier (MID) sequences.

**Additional file 3.** A workflow diagram outlines the steps from nested PCR to bioinformatic analyses (left boxes) and provides corresponding background, reasoning, and details at each step in the process (right boxes).

**Additional file 4.** Six internal quality control samples were PCR amplified and deep sequenced in duplicate. Expected (first column) and actual (sequencing control samples 1-6, averaged across duplicates) haplotype percentages are similar. The average percent error between duplicates was 4.4% (range 0.4-13.6%).

**Additional file 5.** *Pfama1* haplotype sequences and NCBI GenBank accession numbers.

**Additional file 6.** Comparison of haplotype frequencies between all sample types, individual samples, and population cluster samples.

**Additional file 7.** Comparison of demographic factors between sample sequenced individually (n=79) and pooled population cluster samples (N=821) with analysable sequence reads.
