# Supplementary material for: A deep sequencing approach to estimate Plasmodium falciparum complexity of infection (COI) and explore apical membrane antigen 1 diversity
Source: Malar J. 2017 Dec 16;16:490. doi: 10.1186/s12936-017-2137-9 (PMC5732508; doi:10.1186/s12936-017-2137-9)
Supplement: Supplementary file 2 — Additional file 2. Pfama1 heminested primer sequences and Multiplex identifier (MID) sequences. [file 12936_2017_2137_MOESM2_ESM.docx]

| **Primer/MID** | **Sequence (5’→3’)** |
| --- | --- |
| Ama1OF | GCTGAAGTAGCTGGAACTCAA |
| Ama1F^1^ | XXXXXXXXXXCCATCAGGGAAATGTCCAGT |
| Ama1R | TTTCCTGCATGTCTTGAACA |
| MID1 | ACGAGTGCGT |
| MID2 | ACGCTCGACA |
| MID3 | AGACGCACTC |
| MID4 | AGCACTGTAG |
| MID5 | ATCAGACACG |
| MID6 | ATATCGCGAG |
| MID7 | CGTGTCTCTA |
| MID8 | CTCGCGTGTC |
| MID10 | TCTCTATGCG |
| MID11 | TGATACGTCT |
| MID13 | CATAGTAGTG |
| MID14 | CGAGAGATAC |
| MID15 | ATACGACGTA |
| MID16 | TCACGTACTA |
| MID17 | CGTCTAGTAC |
| MID18 | TCTACGTAGC |
| MID19 | TGTACTACTC |
| MID20 | ACGACTACAG |
| MID21 | CGTAGACTAG |
| MID22 | TACGAGTATG |
| MID23 | TACTCTCGTG |
| MID24 | TAGAGACGAG |

^1^Xs represent the 10 nucleotide MID sequence added to the 5’ end of primer Ama1F.
