# Supplementary figures and images for: A deep sequencing approach to estimate Plasmodium falciparum complexity of infection (COI) and explore apical membrane antigen 1 diversity
Source: Malar J. 2017 Dec 16;16:490. doi: 10.1186/s12936-017-2137-9 (PMC5732508; doi:10.1186/s12936-017-2137-9)

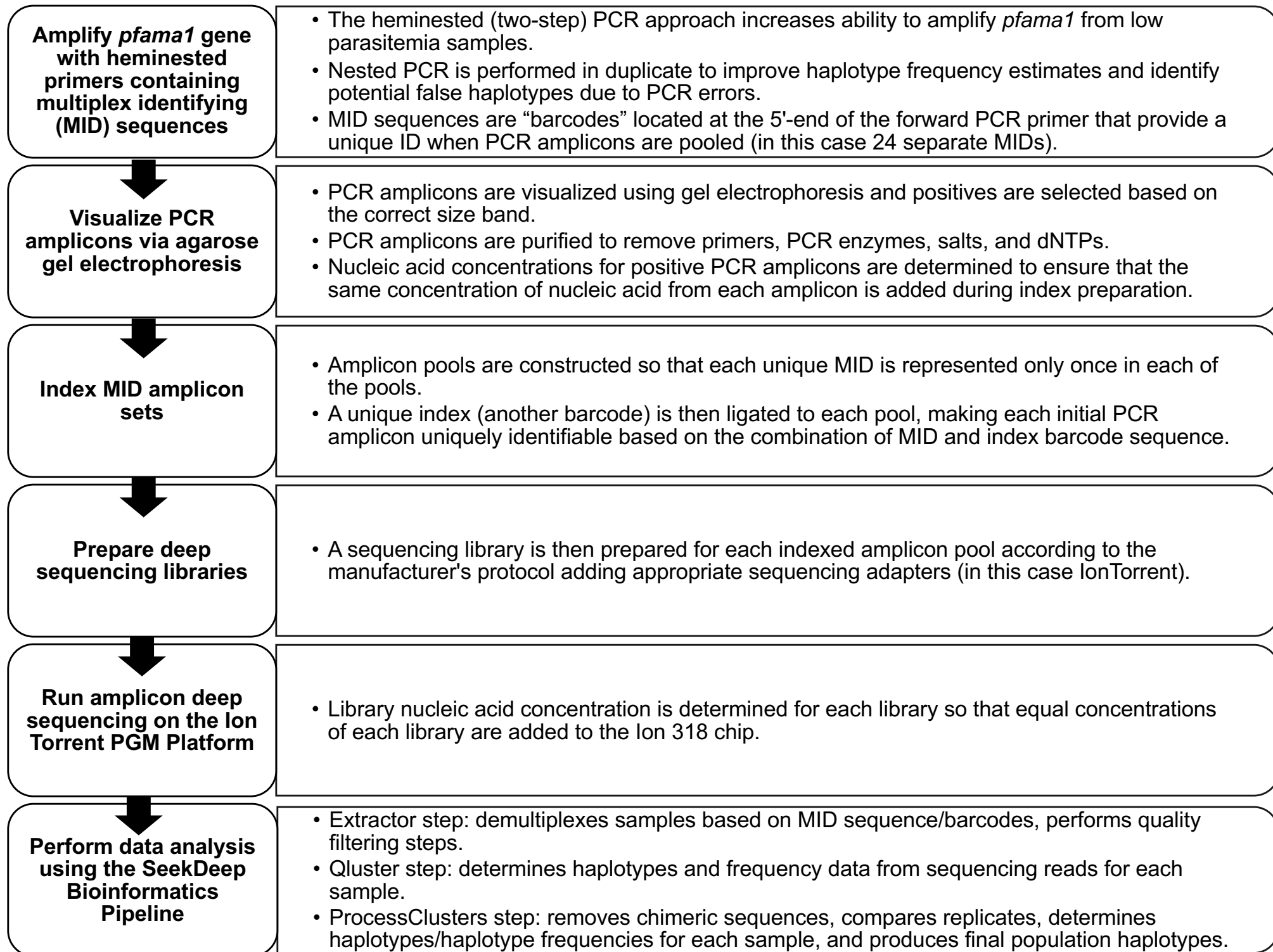

Supplement: Supplementary file 3 — Additional file 3. A workflow diagram outlines the steps from nested PCR to bioinformatic analyses (left boxes) and provides corresponding background, reasoning, and details at each step in the process (right boxes). [file 12936_2017_2137_MOESM3_ESM.pdf]

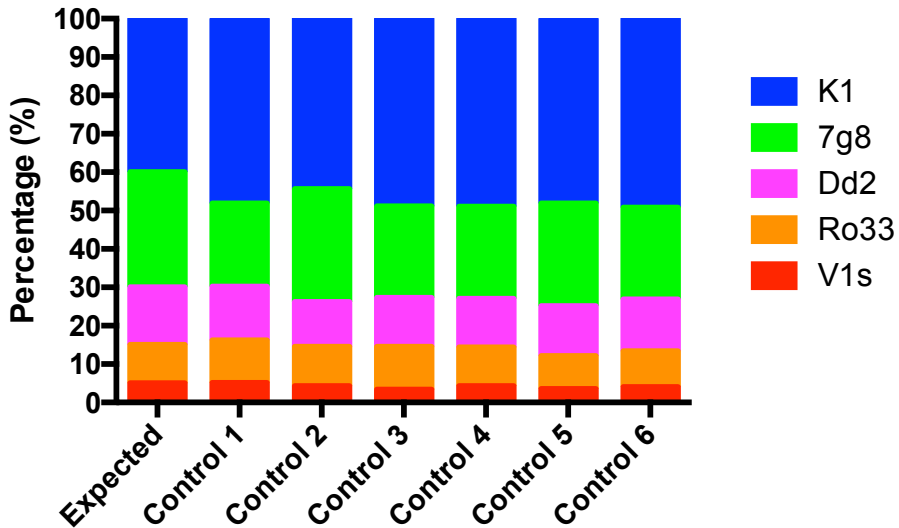

Supplement: Supplementary file 4 — Additional file 4. Six internal quality control samples were PCR amplified and deep sequenced in duplicate. Expected (first column) and actual (sequencing control samples 1-6, averaged across duplicates) haplotype percentages are similar. The average percent error between duplicates was 4.4% (range 0.4-13.6%). [file 12936_2017_2137_MOESM4_ESM.pdf]
