# Supplementary material for: A deep sequencing approach to estimate Plasmodium falciparum complexity of infection (COI) and explore apical membrane antigen 1 diversity
Source: Malar J. 2017 Dec 16;16:490. doi: 10.1186/s12936-017-2137-9 (PMC5732508; doi:10.1186/s12936-017-2137-9)
Supplement: Supplementary file 7 — Additional file 7. Comparison of demographic factors between sample sequenced individually (n=79) and pooled population cluster samples (N=821) with analysable sequence reads. [file 12936_2017_2137_MOESM7_ESM.docx]

| **Variable** | **All Individuals tested**  **N=115** | **All sequenced individuals n=79** | **All pooled individuals**  **N=821** | **Difference between All individuals and Pooled individuals** |
| --- | --- | --- | --- | --- |
| Age, mean (sd) | 33 (10) | 33 (10) | 29 (11) | 4 (p<0.01ƚ) |
| Age range women* | 19-48 | 19-48 | 15-49 | NA |
| Age range men* | 16-58 | 16-58 | 15-59 | NA |
| Sex (% male) | 35 | 33 | 53 | -18 (P<0.01+) |
| Slept under ITN previous night (%) | 5 | 6 | 5 | 0 (p=0.99+) |
| Slept under untreated net previous night (%) | 13 | 13 | 10 | 3 (p=0.23+) |
| % living in urban area | 39 | 41 | 30 | 9 (p=0.05+) |
| Altitude in meters, mean (sd) | 670 (392) | 692 (418) | 654 (342) | 16 (0.23ƚ) |
| * The survey samples men from 15-59 years of age, and women from 15-49 years of age  Ƚ based on two-sample t-test with unequal variances  + based on Pearson’s chi-squared test | | | | |
